# Guidelines on Lifestyle Changes and Breast Cancer—where are we up to and how does this Apply to Black Women with Breast Cancer

**DOI:** 10.1007/s11912-025-01691-1

**Published:** 2025-06-28

**Authors:** Sarah D. Adomah, Susanne Cruickshank

**Affiliations:** 1 Breast Unit, The Royal Marsden NHS Foundation Trust, London, UK; 2 United Kingdom Oncology Nursing Society, London, UK; 3 United Kingdom and Ireland Global Cancer Network, Wilmslow Road, Manchester, M20 4BX UK; 4 Faculty of Health Sciences and Sport, University of Stirling, Stirling, Scotland UK; 5 Division of Clinical Studies, Institute of Cancer Research, London, UK

**Keywords:** Breast cancer, Lifestyle guidelines, Black women, Health disparities, Prevention, Management

## Abstract

**Purpose of Review:**

Breast cancer remains a significant health concern globally, with lifestyle factors playing a crucial role in its prevention and management. This paper aims to summarize the current lifestyle guidelines for breast cancer for consistency and consensus of best practice and to explore their specific implications for Black women with breast cancer.

**Recent Findings:**

There has been a growing body of data from both observational studies and some randomised controlled trials showing the relationship between diet, physical activity, body weight and cancer risk and outcomes. These has led to established national and international guidelines emphasizing the importance of lifestyle advice for survivors in reducing breast cancer risk and a drive for healthcare professionals to guide practice. However, the application and impact of these guidelines can vary across different populations, including Black women, who face unique challenges and disparities in breast cancer outcomes.

**Summary:**

Breast cancer survivors can benefit from counselling about lifestyle changes across the continuum from diagnosis, treatment through to survivorship. Guidelines recommending these changes for breast cancer survivors have been developed to guide healthcare professionals for consistency and best practice. Culturally sensitive approaches and targeted strategies are crucial to effectively reduce breast cancer disparities and improve survival rates in this population.

**Supplementary Information:**

The online version contains supplementary material available at 10.1007/s11912-025-01691-1.

## Introduction

Breast cancer is the most common cancer affecting women and the second most common cancer overall, accounting for 1 in 9 (11.6%) new cancer cases worldwide in 2022. This results in a high burden of disease with approximately, 2.3 million cases, diagnosed in 2022 [1].

In the UK, it is estimated that 1 in 7 women will be diagnosed with breast cancer in their lifetime [2]. The incidence rates are projected to rise by 2% between 2014 and 2035, to 210 cases per 100,000 females by 2035. Notably the incidence of breast cancer appears to be higher among white women compared to black women [2, 3] whereas mortality rate is higher among black women. The population demographics in high-income countries are also changing. In the UK, the numbers identifying as Black, Asian or non-white has continued to rise since the 1991 census, particularly in London, with 46.2% identifying as ethnically non-white in the 2021 census [4]. In contrast, there have been some reports from the USA suggesting rising numbers of breast cancer diagnosis seen in Black, Asian and minority ethnic (BAME) groups approaching parity [5, 6] but a similar trend in poor survival outcomes.

Breast cancer is a universal issue, and the burden of the disease is increasing globally with current estimates indicating that within the next decades, much of the incidence and mortality related will be seen in communities that include members of minority populations or individuals who have experienced health disparities [7]. There are reports of observed high incidence and mortality seen in transitioning countries in Africa, Asia and South America [1, 8]. The change in observed incidence of breast cancer in low-and middle income countries (LMIC) could be multifactorial. Cultural changes and adoption of different lifestyle options influenced by other geographical areas could potentially explain to a certain extent some of the observed changes [8]. Moreover, increased awareness, and removal of disease stigma could potentially result in more women seeking medical advice and therefore more women might access healthcare services and be diagnosed with the disease. However, in the absence of robust data, these potential interpretations should be considered with caution. Another potential reason may be partly due to late-stage diagnosis in LMIC where healthcare systems are fragile and ill-prepared with limited infrastructure to diagnose and manage cancers such as breast, as well as other health inequalities [9–11].

With the implementation of actions to increase early disease detection, and the continual advances in treatment, there is significant improvement in breast cancer outcomes [12]. Yet, breast cancer remains a significant global health concern as it continues to be a leading cause of cancer-related death worldwide [13]. It is imperative therefore that healthcare professionals and stakeholders continue to utilise targeted interventions addressing modifiable risk factors (lifestyle) and improving healthcare access are crucial for reducing the global impact of breast cancer [14, 15].

There has been a growing body of data from both observational studies and some randomised controlled trials showing the relationship between diet, physical activity, body weight and cancer risk and outcomes. These have also indicated benefits of healthy lifestyle change after cancer diagnosis and led to the development of several clinical guidelines with recommendations to inform clinical practice as well as patient education.

Contrarily, studies have consistently reported barriers to healthcare professionals giving such advice. There appears to be a disconnect between healthcare professionals who work within cancer settings and the awareness of these guidelines.

## Background – Black Women and Breast Cancer

Black women with breast cancer have a particularly high risk of death [16] which may be independently associated with personal and social factors, tumour stage, and biological characteristics.

The recent [17] publication highlighted that despite tremendous advances in breast cancer research and treatment over the past three decades—leading to a more than 40% reduction in breast cancer mortality in some high-income countries— there remain inequities, with many groups being systematically left behind, ignored, and even forgotten. Although improvements in mortality rates are seen in White populations, this is not seen in Black women. The incidence among Black women has continued to grow and the mortality rate is disproportionately high [18–20].

In a study of women in the UK aged 15–64 years, South Asian and Black women with breast cancer had 89% and 85% survival rates, compared to a 91% survival rate for white women after 3 years [21, 22]. The NHS Cancer Strategy [23], set out a clear vision for the need to improve cancer survival rates as well as the experience of cancer care across all patient groups, noting poorer outcomes and experience from care in ethnic minority groups.

Many studies have attempted to explore the factors which may be contributing to these disparities. Studies in the UK [24–26] have reported factors such as difficulties organising and attending appointments, other practical and service as well as emotional barriers as having an impact on late disease presentation for ethnic minorities. Other studies have reported feelings of fear, secrecy, cultural beliefs – cancer being viewed as taboo subject and stigma associated with cancer as barriers to accessing and utilising cancer facilities [26, 27]

Some studies have attributed the disparity to increased incidence of adverse biological features, larger and higher-grade tumours with more lymph node involvement, as well as late presentation [28, 29]. Others have reported a lower mean age of diagnosis which may partially explain the incidence of aggressive biological features [5, 30, 31]. Boyer-Chammard et al. [32] and [5], reported that Black women had a higher risk of death, more advanced stage of disease and lower survival than their white counterparts. Howlader et al. [33] also reported that according to SEER data, Black women develop more aggressive breast cancers at earlier ages and have lower survival rates (even by 42%) compared to White women.

Unfortunately, most of the published data exploring these factors derive from USA where access to diagnostic health care and treatment is affected by economic status and may vary between different ethnic groups [34].

There is a gap in data in the UK with few studies focusing on this issue. Nonetheless, the reported outcomes clearly present a trend of higher breast cancer-specific mortality among Black Caribbean and Black African women than White patients [35].

## Lifestyle Changes and Breast Cancer

Much has been written about the importance and impact of lifestyle – diet, obesity, alcohol consumption and physical activity on the incidence and risk of recurrence breast cancer in the literature, including suggesting these changes may influence cancer prevention, disease development, treatment tolerance, and cancer recurrence [13, 14, 36, 37]. Yet a comprehensive review of the literature published in 2014 by the World Cancer Research Fund (WCRF) and International Agency for Research on Cancer [38] (IARC) suggested that there was somewhat limited evidence to guide recommendations on these issues for breast cancer survivors. This in part may be due to inconsistencies among study outcomes and/or methodology. Nonetheless, the WCRF/AICR indicated there was some evidence supporting improved survival among women with breast cancer who have a healthy body weight, are physically active, eat foods containing dietary fibre, soy, or have lower fat or saturated fat intake.

Rock et al. [39] assert that research in breast cancer survivors provides the most substantial and robust body of evidence related to the effects of obesity, physical activity, diet, and alcohol in relation to cancer survival, recurrence, and the risk of second primary cancers among all cancer types.

Among breast cancer survivors, studies have found a consistent link between physical activity and a lower risk of breast cancer recurrence and breast cancer-specific all-cause mortality [40–43]. Physical activity has also been linked to improvements in quality of life, physical functioning, and symptom management such as fatigue. Findings from [44–46] cohort studies have highlighted that a physically active lifestyle after early-stage breast cancer treatment is associated with improved survival.

Recent cohort studies have focused on the potential association between combined lifestyle changes and health outcomes among cancer survivors [47–49] with some results suggesting that making changes are associated with survival benefits [39].

A review by Dieli-Conwright and Orozco [50] on the effect of lifestyle factors on breast cancer mortality concluded that physical activity has the most robust impact of all lifestyle factors on reducing breast cancer recurrence. A recent systematic review by Zhu et al. [51] concluded that adherence to a healthy lifestyle is associated with a lower risk of all-cause mortality (HR 0.57 95% CI: 0.51‒0.65). They noted substantial heterogeneity (*I^2* = 73.58%) across studies reviewed likely due to the differences in studies characteristics such as methodology and definitions of healthy lifestyles among others. In addition, a sub-group analysis demonstrated significant associations between combined lifestyle factors and decreased in mortality in cancer specific sites such as (breast cancer HR = 0.55, 95% CI: 0.48‒0.63) and *I*^2 = 26.97%, as well as cardiovascular disease incidence in cancer survivors. These findings further indicate the potential benefits of adapting to multiple healthier lifestyles behaviours in the long-term management of cancer survivors and promoting our focus in what healthcare professionals should do.

Making positive lifestyle changes can be psychologically beneficial to patients by empowering them, since the feeling of loss of control is one of the biggest challenges of a cancer diagnosis [52]. Kwok et al. [53] describe that many patients with breast cancer seek information from a variety of sources about behaviours that may reduce their risk of recurrence. Conversely, a recent review by Michel et al. [54] reported that lifestyle choices following breast cancer among women in USA was dependant on the recency of their diagnosis. They noted that individuals smoked less, but did not change their alcohol consumption in the five years after diagnosis. However, the diagnosis of breast cancer had a significant negative impact in the amount of exercise possibly because of treatments’ effect on physical ability.

A meta-analysis of 22 prospective cohort studies by Lahart et al. [55] found that breast cancer mortality was significantly reduced among women who reported participating in recreational physical activity after their breast cancer diagnosis (HR 0.59, 95% CI 0.45–0.78). However, there was substantial heterogeneity across the studies reviewed (*I*^*2*^ 57%) which included observational studies such as cohort and case–control studies. The effect was stronger among women who met recommended levels of physical activity as per guidelines, postmenopausal women and women with a BMI greater than 25. Higher lifetime and more recent pre- and post-diagnosis recreational physical activity were significantly associated with a lower risk of all-cause and breast cancer-related death and events.

## Review of Breast Cancer Guidelines

The aim of this commentary is to review available guidance advising on lifestyle changes for breast cancer patients for consistency and consensus of best practice.

Following a literature search, national and international guidelines were reviewed to systematically appraise how professional organizations recommend lifestyle changes and their implementation in clinical practice.

Guidelines for the management of early breast cancer were sourced from the following organisations and reviewed for lifestyle changes recommendations: The National Institute for Clinical Excellence (**NICE, UK**) [56], Associate of Breast Surgery (**ABS, UK**) [57], Scottish Intercollegiate Guidelines Network (**SIGN, UK**) [58], British Association of Surgical Oncology (**BASO, UK**) [59], American Society of Clinical Oncology (**ASCO, USA**) [60], American Cancer Society (**ACS, USA**) [39], Clinical Oncology Society of Australia (**COSA**) [61], Cancer Care Ontario (**CMA**) [62], Canadian Society of Exercise Physiology and American College of Sports Medicine (**CSEP/ACSM**) [63, 64], Canadian Cancer Society (**CCS**) [65], and National Comprehensive Cancer Network (**NCCN, USA**) [66], Dutch Breast Cancer Federation (**DF**) [67], European Society of Medical Oncology (**ESMO**) [68], Australian National Breast and Cancer Centre (**NHMRC**) [61] and World Cancer Research Fund/American Institute for Cancer Research (**WCRF/AICR**) [38].

By mapping against these guidelines, the authors have illustrated the range of guidance provided by different national and international cancer organisations across the world (Table 1) to provide recommendations for good clinical practice.

**Table 1:** Guidelines mapped against lifestyle factors

Guideline	Physical Activity	Diet	Weight Management	Alcohol
ABS	n/a	n/a	n/a	n/a
ACS	✓	✓	✓	✓
ASCO	✓	✓	✓	n/a
BASO	n/a	n/a	n/a	n/a
CCS	✓	✓	n/a	✓
CMA	✓	✓	✓	✓
COSA	✓	✓	✓	✓
CA/NHMRC	✓	✓	✓	✓
DF	✓	✓	✓	✓
ACSM	✓	n/a	n/a	n/a
ESMO	✓	✓	✓	✓
NICE	✓	✓	✓	✓
NCCN	✓	✓	n/a	n/a
SIGN	n/a	n/a	n/a	n/a
WCRF/AICR	✓	✓	✓	✓

A total of 15 national and international guidelines mostly from high-income countries were included in this review. Most of the guidelines reviewed contained roughly similar but not identical recommendations on the lifestyle changes which healthcare professionals may counsel patients with breast cancer about. Hence, worth exploring further and focusing more on the detail of the recommendations.

In 2010 the ACSM [69] developed the first guidelines for cancer survivors suggesting they could safely engage in enough exercise training to improve physical fitness, restore physical functioning, enhance quality of life, and mitigate cancer-related fatigue.

Following on from this, several organisations have developed guideline recommendations to inform healthcare professionals of lifestyle changes to guide practice. However, this review demonstrates there are variations regarding the depth of information provided and areas of lifestyle covered. Whilst some of the guidelines offer detail and specific advice about what lifestyle changes healthcare professionals should advise survivors to adapt such as physical activity, diet, weight management, others appear to be very brief in their descriptions leaving room for misinterpretation.

## Physical Activity

Almost every guideline apart from ABS, BASO and SIGN provided recommendations particularly focusing on physical activity. The **ASCO** [60]**, ACS** [39]**, NICE** [56]**, ACSM** [63, 64]**, ESMO** [68] provide consensus recommendations that healthcare professionals need to share with individuals diagnosed with cancer. These include counselling survivors to be physically active—engage in at least 150 min of moderate-intensity or 75 min of vigorous-intensity activity each week and include strength training exercises at least 2 days per week and maintain a healthy weight. These guideline advocates that patients should be encouraged to incorporate these changes throughout the cancer journey with a strong emphasis on physical activity as a key factor in improving health outcomes for cancer survivors. The **ACS** [39] guidelines further offer resources for nutrition and physical activity counselling.

The DF [67] is the only guideline which defined lifestyle as actions people take to improve/maintain his/her health within the body of the guideline giving context to readers. It further describes some of the benefits of lifestyle changes and breast cancer such as reducing fatigue, anxiety, depression among others. It recommends that lifestyle advice should be a fixed component of aftercare, because a healthy lifestyle reduces the risk of a recurrence and other health complaints and has a positive effect on fatigue, anxiety and depression. Nonetheless, it falls short of expanding on what the most appropriate advice should involve when discussing these changes with patients.

The guideline produced by the ACSM [63, 64, 70] following their roundtable of international multidisciplinary professionals is one of the most detailed to date. They provide a more specific evidence-based exercise prescription to improve common side effects of a cancer diagnosis and treatment, namely anxiety, depressive symptoms, fatigue, health related quality of life, and physical function, along with safety of exercise training in persons with or at risk of breast-cancer related lymphedema. In this guideline, the recommendation indicates the duration, frequency, and/or intensity that healthcare professionals can apply to prescribe physical activities for patients such as 150 min of moderate-intensity aerobic exercise spread over three to five days and resistance training at least two days per week; resistance sessions should involve major muscle groups two to three days per week (eight to 10 muscle groups, eight to 10 repetitions, two sets); and each session should include a warm-up and cool down. But the limitation with this guideline is that it focuses on physical activity mainly with no reference to the other lifestyle aspects.

## Diet and Weight Management

There is good evidence [36, 71] that a healthy diet involving plant-based foods with limited intake of high calorie foods, red meat and processed meats can help prevent cancer reported in the literature. Other observational studies have reported protection against progression of breast cancer by consuming a low-fat/high-fibre diet as well as increased risk of breast cancer recurrence from consuming a “western diet” [72, 73]. But recommendations for cancer survivors are somewhat limited. Perhaps due to insufficient evidence linking diet directly to cancer outcomes [73]. A survey of oncology health professionals found that only half were aware of dietary guidelines for cancer survivors ([74]). Evidently, even though several of the reviewed guidelines (ACS [39], ASCO [60], CCS [65], CMA [75], NCCN [66], NICE [56]) include recommendations for diet and weight management, they appear to be brief statements. They all recommend breast cancer survivors endeavour to eating a healthy dietary pattern that is high in vegetables, fruits and whole grains and low in calorific foods, beverages, and processed meats.

The WCRF/AICR [38], however, describes in detail the recommendations for dietary intake that breast cancer survivors should be encouraged to adopt such as eating foods rich in whole grains, vegetables, fruits and beans and limit consumption of fast foods and other processed foods high in fat, starches, or sugars.

## Alcohol

Like the other lifestyle factors, most of the guidelines reviewed include recommendation regarding alcohol consumption among cancer survivors. The ACS [39], NICE [56], NHMRC [61], ESMO [68], CCS [65], COSA recommends breast cancer survivors limit alcohol intake to reduce breast cancer risk. It is only the ACS which stipulates that women limit alcohol intake to no more than 1 drink per day. These are on the background of different outcomes from studies about alcohol and breast cancer. Several epidemiological studies have identified increased risks of breast cancer outcomes associated with alcohol [76, 77] whereas others have reported no observed association [78, 79].

## Summary

This review and others have highlighted that there are several guidelines with emerging evidence recommending lifestyle changes which will have positive impact on breast cancer survivors available for use with patients following diagnosis. However, physical activity, diet, or lifestyle intervention trials for breast cancer prevention and survivorship among Black and ethnic minority populations are scarce. These existing guidelines are not specific to ethnic groups and the recommendations may not be culturally appropriate in its implementation among these groups. But if we are acknowledging the importance then we need to consider barriers to this information being incorporated into routine care and there is work needed to improve healthcare professionals (HCPs) knowledge and confidence in discussing lifestyle factors.

Perhaps, their rigour of development, mode of implementation and evaluation of impact can be improved in many settings to enable their goal of achieving “best practice” in healthcare. One of the most important knowledge gaps in this direction is the extent to which guidelines affect patient outcomes and how this effect can be enhanced to ensure better care.

A review by Koutoukidis et al. [37] identified a number of such barriers amongst healthcare professionals. These range from perceptions of individual survivors’ current health behaviours, socio-economic factors, and the HCPs-centred barriers about gaps in knowledge and attitudes towards evidence and guideline availability among others. In addition, Anderson et al., [80] commented on other barriers such as lack of service provision, resources for health behaviour change and the promotion of effective interventions within healthcare systems as being suboptimal. They added that often the available data does not appear to represent populations with different backgrounds such as Black African and Caribbean women.

## Conclusion

There is consistent evidence that modifiable lifestyle factors such as physical activity, diet, obesity, alcohol intake is associated with reduced risk for breast cancer-specific and all-cause mortality. Efforts to mitigate against disparities and improve support for Black and other ethnic minority groups will require collaborative approaches and partnerships between multiprofessional agencies, stakeholders, healthcare providers/organizations and communities working with women from diverse backgrounds to ensure local recommendations and implementation are appropriate to their needs in the pursuit for equity in breast cancer care and outcomes.

## Key References

- Delon C, Brown KF, Payne NWS, Kotrotsios Y, Vernon S, Shelton J. Differences in cancer incidence by broad ethnic group in England, 2013–2017. Br J Cancer 2022;126. 10.1038/s41416-022-01718-5.
  - ○ This study is of importance as it highlights the differences in cancer incidence between different ethnic population groups in UK with clear implications for policy and practice, and a renewed focus on the health of minority ethnic groups in the UK who are disproportionately affected.
- Sha R, Kong X, Li X, Wang Y. Global burden of breast cancer and attributable risk factors in 204 countries and territories, from 1990 to 2021: results from the Global Burden of Disease Study 2021. Biomark Res 2024;12:87. 10.1186/s40364-024-00631-8.
  - ○ This study is of importance as one of the biggest study aimed to assess the global burden of breast cancer and identify attributable risk factors across 204 countries and territories from 1990 to 2021.
- Rock CL, Thomson CA, Sullivan KR, Howe CL, Kushi LH, Caan BJ, et al. American Cancer Society nutrition and physical activity guideline for cancer survivors. CA Cancer J Clin 2022;72. 10.3322/caac.21719.
  - ○ This is one of the most comprehensive pieces of work detailing evidence-based guidelines and cancer-specific recommendations for anthropometric parameters - physical activity, diet, and alcohol intake for reducing recurrence and cancer-specific and overall mortality.

## Supplementary Information

Below is the link to the electronic supplementary material.

Supplementary file1 (DOCX 22 KB)

## Data Availability

No datasets were generated or analysed during the current study.
