# Supplementary material for: Guidelines on Lifestyle Changes and Breast Cancer—where are we up to and how does this Apply to Black Women with Breast Cancer
Source: Curr Oncol Rep. 2025 Jun 28;27(8):1031–9. doi: 10.1007/s11912-025-01691-1 (PMC12423215; doi:10.1007/s11912-025-01691-1)
Supplement: Supplementary file 1 — Supplementary file1 (DOCX 22 KB) [file 11912_2025_1691_MOESM1_ESM.docx]

**Supplementary 1 : Mapping of Breast Cancer Guidelines**

| Guideline | **Are Lifestyle Changes Specified** | Definition | Recommendation |
| --- | --- | --- | --- |
| ASCO 2014  ASCO 2015 | Yes –  Obesity  Diet  Physical activity | it classifies this as Health promotion within the  ***Survivorship Care Guideline*** | - counsel survivors to achieve and maintain a healthy weight. - counsel survivors if overweight or obese to limit consumption of high-calorie foods and beverages - increase physical activity to promote and maintain weight loss. - to eat a healthy diet pattern, with adequate macronutrient and micronutrient content from both animal-based and plant-based food options but with a preference to plant-based diet patterns - Caution regarding the overuse and misuse of dietary supplements during and after treatment - Adherence to food safety procedures to avoid foodborne illnesses - Being as physically active as possible |
| American Cancer Society (ACS) 2022 | Yes-  Physical activity, Diet, Alcohol intake | recommendations for reducing recurrence and increasing time to new disease and cancer-specific and overall mortality. | - Engage in at least 150 minutes of moderate-intensity or 75 minutes of vigorous-intensity activity each week. - Include strength training exercises at least 2 days per week - Limit alcohol intake to no more than 1 drink per day for women. |
| ESMO (2023) | Yes –  Diet  Physical activity  Alcohol  Weight management | It refers to general follow-up and psychosocial considerations | - Eat a balanced diet rich in fruits, vegetables, whole grains, and lean proteins.  Limit intake of processed foods and red meat - Maintain a healthy weight to reduce the risk of breast cancer and improve outcomes after diagnosis - exercise regularly - Limit alcohol consumption to reduce breast cancer risk |
| American College of Sports Medicine (2019) | Yes -  Physical activity | Offers duration, frequency, and/or intensity to be recommended. | - 150 minutes of moderate-intensity aerobic exercise spread over three to five days and resistance training at least two days per week - Resistance sessions should involve major muscle groups two to three days per week (eight to 10 muscle groups, eight to 10 repetitions, two sets) - Each session should include a warm-up and cool down |
| NICE 2018, updated 2024 | Yes -  Diet  Physical activity  Alcohol  Weight management | It refers to follow-up considerations of which lifestyle changes and advice is recommended. | - Maintain a healthy weight to lower the risk of breast cancer and improve outcomes after diagnosis - Physical exercise - regular physical activity - Lower alcohol consumption |
| Canadian Cancer Society | Yes -  Diet  Physical activity  Alcohol |  | - Eat a balanced diet rich in fruits, vegetables, whole grains, and lean proteins. - Limit intake of processed foods and red meat - Engage in regular physical activity, aiming for at least 150 minutes of moderate-intensity or 75 minutes of vigorous-intensity exercise per week - Limit alcohol consumption to reduce breast cancer risk. |
| COSA (Clinical Oncology Society of Australia) 2018 | Yes -  Physical activity |  | - At least 150 mins of moderate-intensity or 75 mins of vigorous-intensity aerobic exercise (e.g walking, jogging, cycling, swimming) each week - Two to three resistance exercise (i.e. lifting weights) sessions each week involving moderate-vigorous-intensity exercises targeting the major muscle groups - Exercise recommendations should be tailored to individual’s ability, noting adaptations may be required - Accredited exercise physiologists and physiotherapists are the most appropriate HCPs to prescribe and deliver exercise programs with cancer patients   All health professionals involved in the care of people with cancer **have an important role in promoting these recommendations**. |
| British Columbia (2013) | Yes –  Diet  Physical activity | Healthy living support — for secondary prevention purposes | - patients should be reminded of the importance of a proper diet, - being physically active and maintaining a healthy body weight |
| Dutch Breast Cancer Federation | Yes –  Diet  Physical activity | Lifestyle is defined as: actions people take to improve/maintain his/her health. | - Lifestyle advice is a fixed component of aftercare, because a healthy lifestyle reduces the risk of a recurrence and other health complaints. - Maintain a healthy diet - Regular exercise |
| WCRF/AICR | Yes –  Diet  Physical activity  Alcohol  Weight management |  | - Maintaining a healthy weight - Being physically active - Eating a healthy diet - rich in whole grains, vegetables, fruits, and beans - Limit red and processed meat - Limit sugar-sweetened drinks - Limit alcohol consumption - Avoid weight gain during adulthood |
| Canadian Medical Association (CMA, 2005) | Yes -  Weight management |  | - Weight management should be discussed with all breast cancer survivors - Overweight patients should be encouraged to participate in evidence-based weight-management programs |
| NCCN 2020 | Yes –  Diet  Physical activity  Food safety  Supplements |  | - Recommendations to eat a healthy diet pattern, with adequate macronutrient and micronutrient content from both animal-based and plant-based food options but with a preference to plant-based diet patterns - Caution regarding the overuse and misuse of dietary supplements during and after treatment - Adherence to food safety procedures to avoid foodborne illnesses - Being as physically active as possible |
